# Parameter Estimation of the Thermal Network Model of a Machine Tool Spindle by Self-made Bluetooth Temperature Sensor Module

**DOI:** 10.3390/s18020656

**Published:** 2018-02-23

**Authors:** Yuan-Chieh Lo, Yuh-Chung Hu, Pei-Zen Chang

**Affiliations:** 1 Institute of Applied Mechanics, National Taiwan University, No. 1, Sec. 4, Roosevelt Rd., Taipei 10617, Taiwan; r04543023@ntu.edu.tw (Y.-C.L.); changpz@ntu.edu.tw (P.-Z.C.); 2 Department of Mechanical and Electromechanical Engineering, National ILan University, No.1, Sec. 1, Shennong Rd., Yilan City, Yilan County 260, Taiwan

**Keywords:** Bluetooth temperature sensor module, machine tool spindle, parameter estimation, predictive thermal characteristic, thermal network model, system identification

## Abstract

Thermal characteristic analysis is essential for machine tool spindles because sudden failures may occur due to unexpected thermal issue. This article presents a lumped-parameter Thermal Network Model (TNM) and its parameter estimation scheme, including hardware and software, in order to characterize both the steady-state and transient thermal behavior of machine tool spindles. For the hardware, the authors develop a Bluetooth Temperature Sensor Module (BTSM) which accompanying with three types of temperature-sensing probes (magnetic, screw, and probe). Its specification, through experimental test, achieves to the precision ±(0.1 + 0.0029|t|) °C, resolution 0.00489 °C, power consumption 7 mW, and size Ø40 mm × 27 mm. For the software, the heat transfer characteristics of the machine tool spindle correlative to rotating speed are derived based on the theory of heat transfer and empirical formula. The predictive TNM of spindles was developed by grey-box estimation and experimental results. Even under such complicated operating conditions as various speeds and different initial conditions, the experiments validate that the present modeling methodology provides a robust and reliable tool for the temperature prediction with normalized mean square error of 99.5% agreement, and the present approach is transferable to the other spindles with a similar structure. For realizing the edge computing in smart manufacturing, a reduced-order TNM is constructed by Model Order Reduction (MOR) technique and implemented into the real-time embedded system.

## 1. Introduction

The thermal characteristics of machine tool spindles play an important role in the development of high-precision and high-speed machining. Many literatures about the machining error analysis had indicated that a large amount of the total machining errors attributed to the thermal issue [1,2]. In recent years, diagnostics and prognostics of sudden failure of machine tool spindle have drawn considerable attention. Hence, the model-based method is the key technology for predicting the thermal behavior of spindle. Bossmanns and Tu et al. developed a comprehensive thermo-mechanical model to characterize the heat transfer and quantitative power flow of a high-speed motorized spindle based on Finite Difference Method (FDM) [3,4,5]. On the other hand, Finite Element Method (FEM) has the advantage of fully simulating the thermo-dynamic-mechanical behavior of the spindle. These FEM models not only simulate the temperature distribution and the thermal deformation of machine tool, but also take the bearing stiffness and thermal contact resistance into consideration [6,7]. Experimental and simulation evidence indicated that the temperature distribution of spindle is axisymmetric [8]. Meanwhile, the thermal resistance network model was proposed considering the thermal contact resistance based on fractal and Hertz contact theory [9,10]. Several relevant issues were widely investigated in thermal characteristics of machine tool spindle, such as thermal contact resistance, lubricant viscosity, cooling condition, thermally induced preload, and transient thermal behavior [6,11,12,13]. Heat flux is one of the most important parameters of the thermal behavior of spindle. The heat flux of a thermal system could be inversely predicted by recursive least squares algorithm from the thermal resistance network model [14] or finite element model [15]. System identification techniques have drawn much attention to many thermal predictive and control applications, such as the core temperature real-time estimation for lithium ion batteries [16], the temperature prediction of the squirrel-cage rotor of induction motor [17], the thermal system analysis of buildings [18], and the transient thermal impedance of semiconductor [19]. Regarding the failure prevention of permanent magnet synchronous motors, the grey-box system identification of the model-based lumped-parameter thermal networks (LPTNs) has been developed to estimate the permanent magnet and winding temperature [20,21,22].

This article presents a simplified and reduced-order thermal model for a machine tool spindle, which can predict the thermal response of certain crucial points within the spindle. Though FEM and FDM are great approaches to analyze the thermal characteristic of the spindle; however, they require a large amount of numerical computation. These methods are unsuitable for in-situ application due to their high demand of numerical computation. Instead of comprehensively analyzing the heat transfer equations, this article establishes a lumped-element Thermal Network Model (TNM) that effectively reduces the computation load while retaining the predictive performance. The TNM consists of the parameters of thermal resistances, thermal capacitances, and heat sources. The assumptions and simplifications of those parameters are made based on the theory of heat transfer and empirical formula. Note that the global and local solutions of the parameters are solved from the measured temperature data by means of nonlinear least square method and grey-box system identification method. The TNM is verified by self-validation as well as external-validation. Moreover, the Short-Circuit Time Constant (SCTC) method is applied to approximate the low-frequency band limitation. Also, the Model Order Reduction (MOR) techniques allow for reducing the order of the estimated model while retaining the performance. Furthermore, Bluetooth Temperature Sensor Module (BTSM) is developed that provides high accuracy, high stability, and five-channel temperature measurement. Three types of temperature sensing probes (magnetic, screw, and probe) based on Resistance Temperature Detector (RTD) are constructed in this work, those are suitable for being placed at some characteristic locations such as into the outer-ring of bearings, approaching to the spindle, and on the surfaces of the housing.

## 2. Theoretical Background 

### 2.1. Thermal Characteristics of Spindle

Many researchers have comprehensively investigated the thermal characteristics of machine tool spindle, including heat generation, forced heat convection over rotating shaft and annulus, heat conduction, heat radiation, and heat capacity. The primary concern is the speed-related influence, hence, the degree of each leading term related to the rotating speed should be determined especially. The frictional torque of the angular contact ball bearings causes the major heat generation of the externally driven spindle. Harris proposed that the heat sources were mainly caused by three factors: applied load (*Q_l_*), viscous shear (*Q_v_*), and spinning motion (*Q_s_*) [23].

(1) $$Q_{f}=Q_{l}+Q_{v}+Q_{s}=1.047\times 10^{-4}\times [n(M_{l}+M_{v})+\sum_{j=1}^{Z}n_{si}M_{si,j}+n_{so}M_{so,j}]$$

where *M_l_* is the applied-load-dependent term, *M_v_* is the viscous-shear-dependent term, and *M_si_* and *M_so_* are the spinning-motion-dependent terms, n denotes the rotational speed of bearing, *n_si_* and *n_so_* denotes the spin speeds of the inner and outer rolling element, respectively, and *Z* is the number of rolling elements.

Stein and Tu modified Palmgren’s equation for specifying the thermally induced preload of bearing [24]. The bearing friction torque due to applied load (*M_l_*) is obtained by Equation (2). In their research, the predicted thermally induced preload would reach steady over time and its magnitude grew with the rotational speed. The nonlinear relationship between thermally induced preload and rotational speed is like the power function.

(2) $$M_{l}=\mu_{l}f_{l}(F_{n}+F_{t})(r_{i}+r_{b})$$

where *F_t_* is the thermally induced preload, *F_n_* is the contact force from external load, *r_i_* and *r_b_* is the radius of inner ring and rolling element, *μ_l_* is the friction coefficient due to load. The empirical Equation (3) explains the frictional torque that is caused by lubricant shear viscosity (*M_v_*) within the bearing.

(3) $$\begin{array}{l} M_{v}=10^{-7}f_{0}{\left(\nu_{L}n\right)}^{2/3}d_{m}{}^{3},\;\mathrm{for}\;\nu_{L}n\geq 2000\\ M_{v}=160\times 10^{-7}f_{0}d_{m}{}^{3},\;\mathrm{for}\;\nu_{L}n<2000 \end{array}$$

where *f*_0_ is a factor depending on bearing type and lubrication, *ν_L_* is lubricant kinematic viscosity. For angular contact ball bearing, the gyroscopic moment of the rolling elements necessarily leads to boring motion that would cause the friction torque on the contact surface, and thus also introduce heat generation. The friction torque related to spinning motion (*M_s_*) is obtained by Equation (4) [25].

(4) $$M_{s}=\frac{3\mu_{s}F_{roll}ae}{8}$$

where *F_roll_* is the contact load, *a* represent the major axes of the elliptical contact area, *e* is the elliptical integral of the second kind, and *μ_s_* is the friction coefficient.

The primary concern is to evaluate the heat source within the spindle thermal system associated with the spindle rotational speed (*ω*). Many researchers had indicated that the viscous friction has the influential contribution in total frictional torque within the bearing [12]. As for simplification, due to the power function trend of thermally induced preload influence, the polynomial-like function might have the ability to predict the heat generation of bearing. As mentioned above, substituting Equations (2)–(4) into Equation (1), the authors conclude that the heat source can be expressed as a function of the viscous-related term (*ω*^5/3^) and the remaining term (*ω*), as described in Equation (5).

(5) $$\begin{array}{l} Q_{f}\sim f(\omega ,F_{t})\overset{\;(1)\;}{\to }f(\omega^{5/3},\;\omega^{1})\\ {}_{\left(1\right)}F_{t}\sim f(\omega^{c_{0}}) \end{array}$$

An analytical solution describes the forced convective heat transfer over rotating shaft [26]. The average Nusselt number ($\bar{\mathrm{Nu}}$), Reynolds number (Re), and Prandtl number (Pr) are determined by the following equations.

(6) Nu¯=0.6366(RePr)1/2

(7) Re=ωd22νair,Pr=νairαair

Assume constant material properties, the rotational speed dominates the forced heat convection. This implies that the forced convection coefficient is a speed-related term being proportional to *ω*^0.5^. In this case, we take the rotational speed as the leading term and express the thermal resistance of forced convection around moving surface as a function of *ω*^−0.5^.

(8) hforced=Nu¯×kaird∝Re0.5∝ω0.5

(9) $$R_{forced}=\frac{1}{h_{forced}A}\sim f(\omega^{-0.5})$$

The forced heat convection over the surfaces of rotating annulus can be determined by the following Equations (10) [27]. Taylor number (Ta) and the geometry factor (*F_g_*, *P*) are obtained. Note that the geometry factors (*Fg* and *P*) depend on the inner and outer radius of annulus only; therefore, it can be viewed as a constant value.

(10) $$\bar{\mathrm{Nu}}=0.409{\left(\frac{{\mathrm{Ta}}^{2}}{F_{g}}\right)}^{0.241},{\;\mathrm{for}\;10}^{4}<\frac{{\mathrm{Ta}}^{2}}{F_{g}}<10^{7}$$

where

$$\mathrm{Ta}=\frac{\omega {\left[\frac{\left(r_{o}+r_{i}\right)}{2}\right]}^{0.5}{\left(r_{o}-r_{i}\right)}^{1.5}}{\nu };F_{g}=(\frac{\pi^{4}}{1697}){\left(1-\frac{\left(r_{o}-r_{i}\right)}{\left(r_{o}+r_{i}\right)}\right)}^{-2}P^{-1};$$

$$P=0.0571\{1-0.652(\frac{r_{o}-r_{i}}{r_{i}})\}+0.00056{\left\{1-0.652(\frac{r_{o}-r_{i}}{r_{i}})\right\}}^{-1}$$

and *r_i_* and *r_o_* are the inner and outer radius. Namely, the coefficient of forced heat convection can be derived in Equation (11), which is proportional to *ω*^0.482^. Thus, it is appropriate to formulate the thermal resistance of forced convection near annulus as a function of *ω*^−0.482^ in Equation (12).

(11) $$h_{annulus}\propto \bar{\mathrm{Nu}}\propto {\mathrm{Ta}}^{0.482}\propto \omega^{0.482}$$

(12) $$R_{annulus}=\frac{1}{h_{annulus}A_{annulus}}\propto \omega^{-0.482}$$

According to [28], free convection around a horizontal cylinder and stationary ambient can be estimated by the following Equation (13).

(13) $$\bar{\mathrm{Nu}}={\left\{0.6+0.387{\left[\frac{\mathrm{Gr}\mathrm{Pr}}{{\left[1+{\left(0.559/\mathrm{Pr}\right)}^{9/16}\right]}^{16/9}}\right]}^{1/6}\right\}}^{2}\;for\;10^{-5}<\mathrm{Gr}\mathrm{Pr}<10^{12}$$

where

$$\mathrm{Pr}=\frac{\nu_{air}}{\alpha_{air}};\;\mathrm{Gr}=\frac{g\beta (T_{s}-T_{a})D^{3}}{\nu_{air}^{2}}\overset{\left(1\right)}{=}\frac{g(T_{s}-T_{a})D^{3}}{T_{f}\nu_{air}^{2}}\;{}_{\left(1\right)}\beta \sim \frac{1}{T_{f}}(idea\;gas\;approximation);\;T_{f}=\frac{T_{s}+T_{a}}{2}$$

Except for the material properties and geometry factor, the free convection coefficient is dependent on the spindle surface temperature (*T_s_*) and ambient temperature (*T_a_*) particularly. It would be difficult to simulate the free convective phenomenon according to the temperature difference. Alternatively, the rotational speed has strong correlation to the temperature difference. We assume that the free convective coefficient can be expressed as the function in Equation (14). Hence, the free convective thermal resistance can be derived as two terms, a constant term and a speed-related term. Especially, all of the assumptions must be verified by experimental results, and it will be further discussed in Section 5.

(14) $$\begin{array}{c} h_{free}=h_{free,c}+h_{free,s}\omega^{n}\\ R_{free}=\frac{1}{h_{free,c}A+h_{free,s}\omega^{n}A}=R_{free,c}∥R_{free,s} \end{array}$$

In general, the radiative thermal resistance can be expressed as Equation (15). It indicates that the resistance of thermal radiation depends on surface temperature (*T_s_*) and ambient temperature (*T_a_*). Due to the extremely tiny value of Stefan-Boltzmann constant (*σ*) and the emissivity (*ε*), the influence of surface temperature variation would be limited. Consequently, the radiative thermal resistance can be considered as a constant value, which would be further verified by experiment.

(15) $$R_{rad}=\frac{1}{\varepsilon \sigma A(T_{s}^{2}+T_{a}^{2})(T_{s}+T_{a})}$$

The thermal capacitance is directly determined by the element properties (volume, heat capacity, density) through Equation (16). It represents the store of energy that is required to increase the temperature. In other words, the thermal capacitance value is determined once the spindle structure is designed in practice. Thereby, it is assumed as a constant, when considering that the material properties remain relatively the same under different operating conditions. The estimated value of each thermal capacitance has the decisive influence on the thermal time constant.

(16) $$C=\rho Vc_{p}$$

### 2.2. System Identification Technique

**Figure 1:**
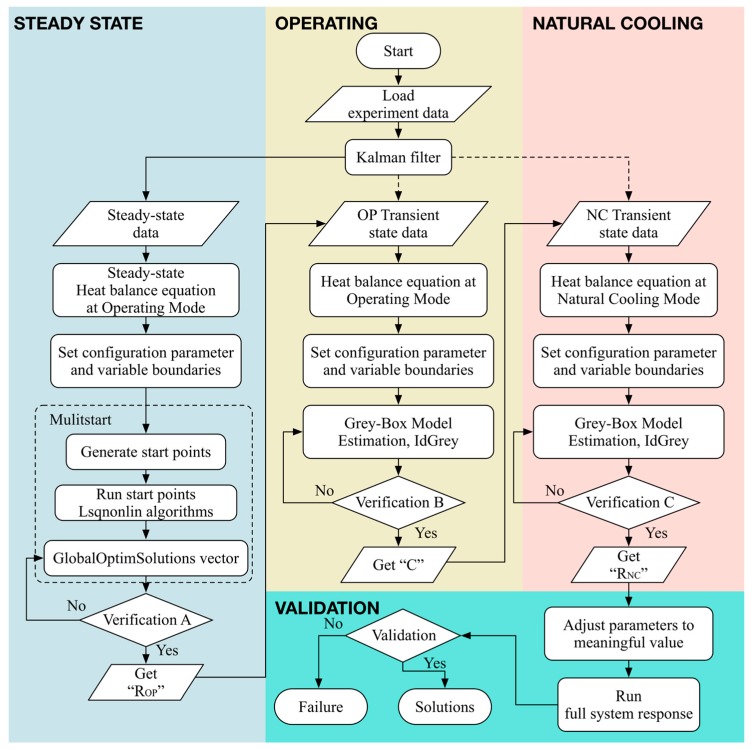
System identification procedure.

The TNM of machine tool spindle is identified by system identification methodology based on physical concept, namely the so-called grey-box estimation. It is utilized to predict the temperature distribution of spindle. Figure 1 explains the system identification procedure for the TNM of machine tool spindle comprehensively. First, through the temperature data measured at steady state, solve for the thermal resistances of TNM at operating mode by means of the “lsqnonlin” function and the “trust-region-reflective” algorithm; the aforesaid function and algorithm are the nonlinear least square regression provided by MATLAB [29]. Second, solve for the thermal capacitance of TNM by means of the “greyest” function provided by MATLAB; that function is exactly the grey-box model identification [29]. Finally, the thermal resistances at natural cooling mode are obtained by the grey-box model identification as well. The least square system identification problem usually exists non-unique solutions satisfying the cost function (minimization). It is expected that some irrational solutions can be eliminated based on physical sense, for example, the convective thermal resistance is generally greater than the conductive one. Furthermore, it must be emphasized that while inversely estimate the heat generation, thermal resistance, and thermal capacitance at the same time, some analogous solutions might occur because different solutions with the same ratio among all of the parameters might introduce the same result. Fortunately, the total thermal capacitance ($\overset{4}{\underset{j=1}{\sum }}C_{j}$) is a constant value; thus, the appropriate solution can be determined accordingly. Finally, it is necessary to verify the performance of the estimated TNM; hence, the self-validation and external validation are further investigated in Section 5.

## 3. Parameterization Methodology for the Thermal Network Model of Spindle

### 3.1. Parameterization Strategy

The assumptions for modeling the TNM are listed as follows:

(1) lumped element model assumption is valid, Bi << 0.1;
(2) temperature distribution of spindle is axisymmetric;
(3) heat generation and forced convective resistance are assumed according to the theoretical and empirical Equations (5), (9), and (12);
(4) heat transfer through radiation and conduction are considered as a constant thermal resistance, including thermal contact resistance; and,
(5) free convection coefficient is assumed as a function of $h_{free,c}+h_{free,s}\omega^{-0.5}$.

According to previous research, the temperature distribution of spindle is almost axisymmetric. For this reason, the two-dimensional heat equation with internal heat generation can be implemented to describe the three-dimensional thermal behavior. Furthermore, based on the assumption of lumped element model, the TNM can characterize the heat transfer inside the spindle.

### 3.2. Estimated Thermal Network Model in State-Space

While the spindle operates at a constant rotational speed, the frictional heat generation are assumed to be a constant source, denoted as *Q_f_*_1_, *Q_f_*_2_. As a result, the heat balance equation with internal heat generation is expressed as

(17) $$\left[C]\frac{\partial \{T\}}{\partial t}=[K]\{T\}+[K_{a}]\{T_{a}\}+\{Q_{H}\right\}$$

The authors define the temperature difference (*θ*) with respect to the ambient temperature.

(18) $$\{\theta (t)\}\equiv \{T(t)\}-T_{atm}$$

Substituting it into Equation (17) gives the heat balance equations

(19) $$\left[C]\frac{\partial \{\theta \}}{\partial t}=[K]\{\theta \}+\{Q_{H}\right\}$$

**Figure 2:**
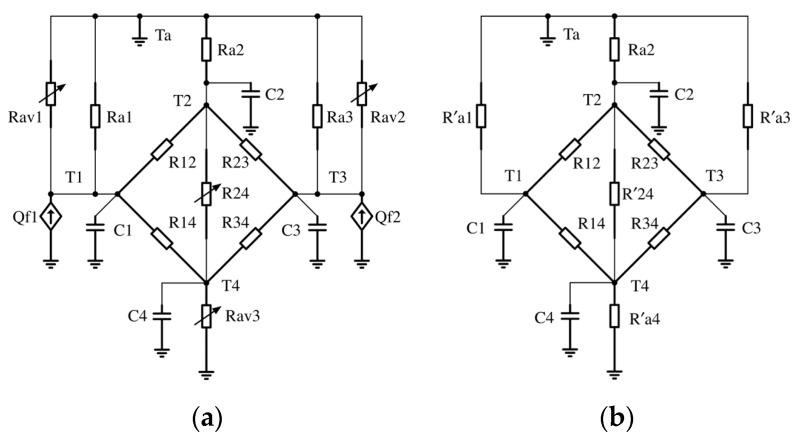
(**a**) 4-node Thermal Network Model (TNM) at operating mode; (**b**) 4-node TNM at natural cooling mode.

Consider the physical insight and geometry of the spindle; we construct the lumped-parameter TNM of the spindle with four nodes, illustrated in Figure 2, which are rear bearing A (*T*_1_), midpoint of housing (*T*_2_), front bearing D (*T*_3_), midpoint of shaft (*T*_4_), respectively. The specific locations will be illustrated latterly in Section 4.2. TNM contains four types of components, thermal resistance, speed-related thermal resistance, thermal capacitance, and heat source. The authors classify the thermal behavior of spindle into two modes, operating mode and natural cooling mode. At operating mode, the spindle is operated at a constant rotation speed and the initial temperature is equal to the ambient temperature, illustrated in Figure 2a. At natural cooling mode, the spindle is stopped and releases the heat that is stored during operating mode, as illustrated in Figure 2b. The state-space representation of the TNM at operating mode is expressed as

(20) $$\{\begin{array}{c} \dot{x}(t)=A_{op}x(t)+B_{op}u(t)\\ y(t)=C_{op}x(t)+D_{op}u(t) \end{array}$$

According to the assumptions, the convective resistance coefficient (*r_av_*_j_, *r*_24_) and the heat generation coefficient (*q_f_*_1_–*q_f_*_4_) are unknown parameters needed to be solved.

(21) $$\{\begin{array}{l} R_{avj}=r_{avj}{\left(\omega /\omega_{0}\right)}^{-0.5},\;R_{24}=r_{24}{\left(\omega /\omega_{0}\right)}^{-0.482}\\ Q_{f1}=q_{f1}{\left(\omega /\omega_{0}\right)}^{5/3}+q_{f3}{\left(\omega /\omega_{0}\right)}^{1}\\ Q_{f2}=q_{f2}{\left(\omega /\omega_{0}\right)}^{5/3}+q_{f4}{\left(\omega /\omega_{0}\right)}^{1} \end{array}$$

where *ω*_0_ is 5000 rpm, thus the unit of the coefficients can be meaningful. At steady state, the thermal capacitances can be ignored due to the temperature derivative terms decay at steady state. Therefore, rewriting the heat balance equation as:

(22) $$A_{std}\theta +B_{std}\theta \omega^{0.482}+C_{std}\theta \omega^{0.5}+D_{std}\omega^{5/3}+E_{std}\omega^{1}=0$$

Equation (22) is a Single-Input-Multiple-Output (SIMO) system whose input is the spindle rotation speed (*ω*) while the spindle temperatures (*θ*) are the outputs. As the speed-related parameters varying with different spindle rotation speeds, the thermal response of TNM would change as well; therefore, these nonlinear behaviors make the TNM more complicated. Likewise, the state-space representation of the TNM at natural cooling mode is expressed as:

(23) $$\{\begin{array}{cc} \dot{x}(t)=A_{nc}x(t)+B_{nc}u(t), & x_{i}(0^{-})=\theta_{i}(0^{-})\\ y(t)=C_{nc}x(t)+D_{nc}u(t) & \end{array}$$

The specific system parameter matrices are shown in Appendix.

## 4. Experiment Setup

### 4.1. Self-Made Bluetooth Temperature Sensor Module

**Figure 3:**
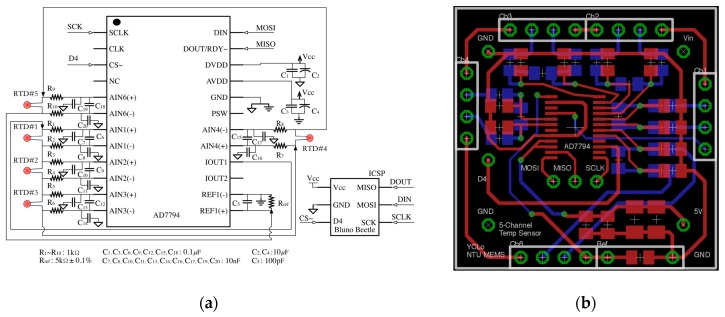
(**a**) Circuit diagram of Bluetooth Temperature Sensor Module (BTSM); (**b**) Printed Circuit Board (PCB) layout of BTSM.

**Figure 4:**
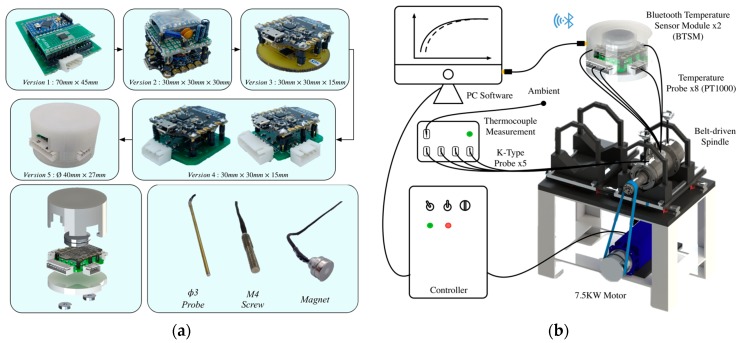
(**a**) Evolution of BTSM and three types of temperature probes; (**b**) Experimental setup.

The performance (accuracy, resolution, stability…) of the sensor is one of the key points directly affecting the quality of the system identification results. Due to the high stability and accuracy of RTD, the authors develop a BTSM that provides multi-channel temperature measurement with high precision. Figure 3a shows the circuit design of BTSM, it is implemented with a Bluno Beetle [30] as the microcontroller, a 24-bit AD7794 [31] as the analog-to-digital converter, a reference resistor with low temperature coefficient of resistance, 4-wire Kelvin measurement, and the Printed Circuit Board (PCB) Layout (Figure 3b). After several versions of evolution, the BTSM (Figure 4a) has achieved with high accuracy of ±(0.1 + 0.0029|*ϑ*|) °C, the resolution of 0.00489 °C, and the module size is only Ø40 mm × 27 mm. The specification is listed in Table 1. As the front-end temperature acquisition element, three types of temperature probe (screw type, magnetic mount type, and probe type) are fabricated by platinum RTD (PT1000) and sealed with thermal conductive Gel (Figure 4a). Magnet type probe is made for surface temperature measurement, and the other types are made for the shaft, housing, bearing temperature measurement. The fabrication process of the temperature probe is shown in supplementary materials Figure S1. It should be mentioned here that, to improve the precision of the BTSM, the Kalman filter is used to filter the noise from the measured temperature signal.

### 4.2. Experiment Setup

**Figure 5:**
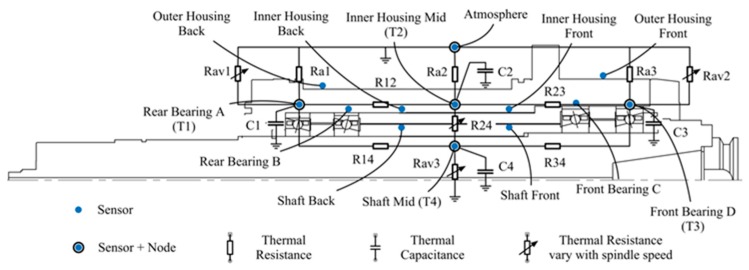
Sensor location and TNM representation in machine tool spindle.

For validating the commercial application of the present TNM and BTSM, the authors purchased a customized, but without loss of generality, spindle warm-up system. Illustrated in Figure 4b, it contains a spindle driven by a 7.5 kW motor through belt, a controller accompanying with a PC being used to conduct the programmable control of motor, a commercial thermocouple device being used to verify the feasibility of the self-made BTSM, etc. The embedded locations of the temperature probes are designed based on the thermal characteristic of machine tool spindle. Specifically illustrated in Figure 5, the temperature sensors are strategically located at 12 nodes near the front and rear bearings, spindle housing, shaft, spindle surface, and ambient temperature, respectively.

## 5. Results

**Figure 6:**
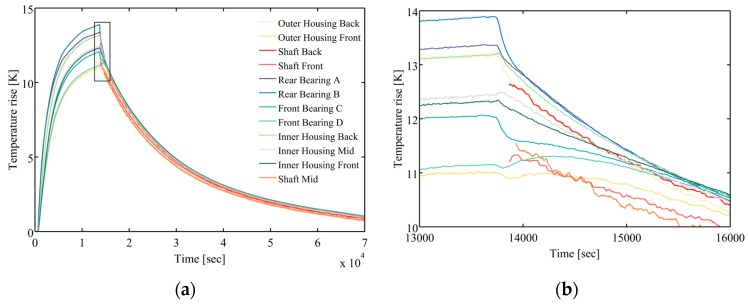
(**a**) Experimental result of 12 nodes on 6021 rpm; (**b**) The transition region of reaching thermal equilibrium.

The transient temperature distribution of spindle is measured by BTSM at various rotation speeds. Figure 6a shows a typical temperature rise curves at the rotation speed of 6021 rpm. Note that, at operating mode is similar to the charging mode of R-C circuit, more specifically, the zero-state response. Likewise, the temperature fall behavior at the natural cooling mode is similar to the discharging mode of R-C circuit, more specifically, the zero-input response. Turn off the spindle right after reaching its steady state, meanwhile insert the temperature probe into the shaft. Consequently, the complete steady-state temperature distribution of spindle can be measured. Zoom in the region of the rectangular frame line of Figure 6a, namely the transition from operating mode to natural cooling mode, Figure 6b reveals that the uneven temperature distribution accumulated at operating mode will gradually reach the thermal equilibrium in nearly 1000 s. After that, the entire spindle behaves relatively as a homogeneous temperature element and cools through free heat convection and radiation heat transfer. It implies that the thermal convective resistances are greater than the thermal conductive resistances. As a result, it further proves that the lumped element assumption is valid. Following the system identification procedure, as mentioned in Section 2.2, the estimated parameters of the TNM are listed in Table 2 and Table 3, and the value of the thermal parameters are calculated at the rotational speed of 5000 rpm.

**Figure 7:**
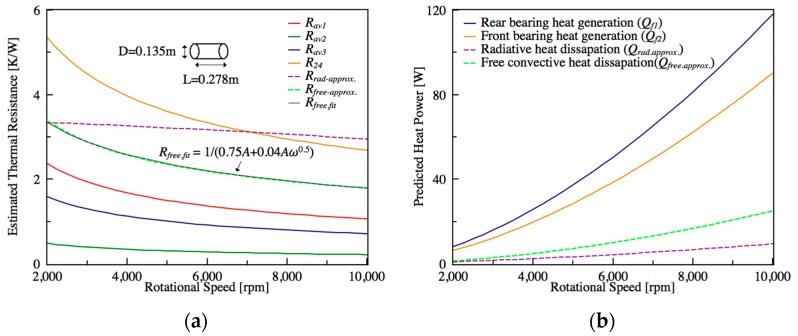
(**a**) Simulated heat generation varying with rotational speeds; (**b**) Estimated convective resistance varying with rotational speeds.

To verify the assumptions of the free convective and radiative heat transfer, the measured temperature of rear bearing (*T*_1_) at steady state is used to simulate *h_free_* and *h_rad_*. Due to the temperature of rear bearing is the highest one among all of the other positions, the simulation results can indicate the significant influence. In Figure 7a, the approximate computation of *R_rad_* (the purple dashed line) from Equation (15) is almost a constant value. As for free convective heat transfer coefficient, the authors consider the spindle to be a homogeneous cylinder with the diameter (D) of 0.135 m and length (L) of 0.278 m, and its other material properties are based on 25 °C. Consequently, the approximately calculated *R_free_* (green dotted line) from Equation (13) can be curve-fitted with the formula $1/A\left(h_{free,c}+h_{free,s}\omega^{-0.5}\right)$. For this reason, it is appropriate to adopt these assumptions. Figure 7b shows the predicted heat generations of rear and front bearings. One can estimate the dissipated heat through radiation (*Q_rad.approx._*) and free convection (*Q_free.approx._*) by means of the preceding approximated *h_free_* and *h_rad_*. Furthermore, Figure 7a shows the speed-dependent parameters with respect to the rotational speed. It indicates that the nonlinear behavior is more significant under lower rotational speed. For this reason, it is necessary to take the speed-dependent thermal behavior into consideration. In other words, due to the varying of the thermal parameters, the poles of the estimated TNM would slightly change with different operating speeds, which will be discussed latterly in Section 5.4.

### 5.1. Steady State Self-Validation

**Figure 8:**
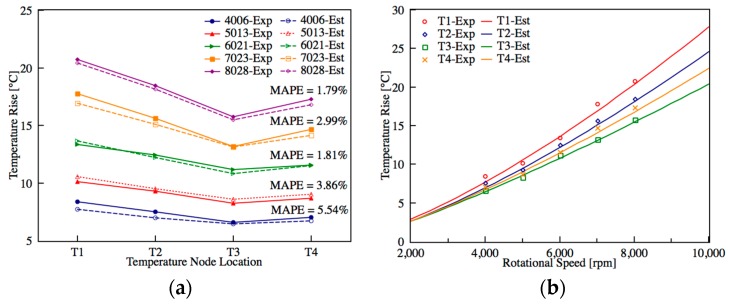
(**a**) Self-validation between predicted and measured steady state temperature, *T*_1_ to *T*_4_ are the positions of the rear bearing A, midpoint of inner housing, front bearing D, and midpoint of shaft, respectively; (**b**) Steady-state temperature validation.

After applying the nonlinear least square estimation to find the minimum 2-norm solution of the overdetermined system in state space representation, the global solutions at steady state can be found, which represent the sets of the optimized thermal resistance value. First, the model is self-validated at steady state through a mutual comparison of the predicted and measured temperature at five different rotation speeds, say 4006, 5013, 6021, 7023, and 8028 rpm, as shown in Figure 8a. The predicted and measured temperatures agree very well with each other, and the maximum mean average percentage error (MAPE) is only 5.54%. Moreover, it should be emphasized that the heat generation and steady state temperature are closely related, which is confirmed from the similarity of Figure 7b and Figure 8b.

### 5.2. Transient State Self-Validation

**Figure 9:**
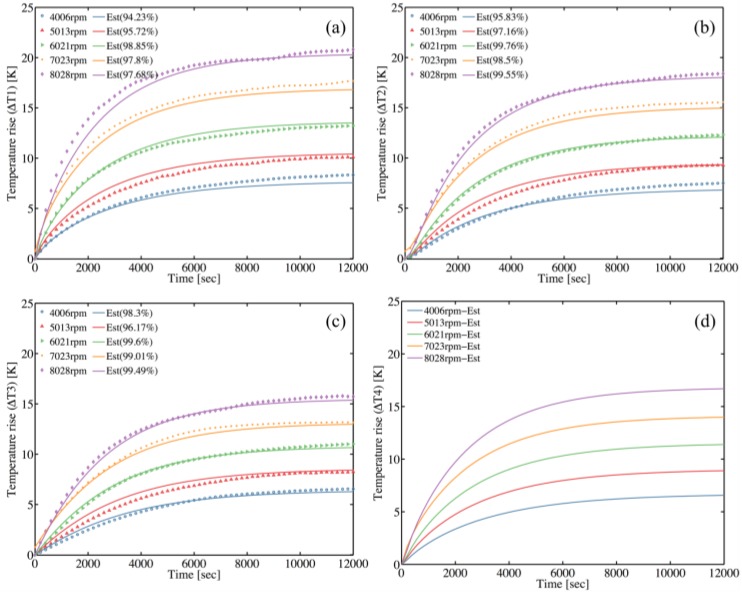
Model self-validation at operating mode with various rotation speeds (**a**) Rear bearing (*T*_1_) temperature validation (**b**) Midpoint of housing (*T*_2_) temperature validation (**c**) Front bearing (*T*_3_) temperature validation (**d**) Midpoint of shaft (*T*_4_) temperature validation.

**Figure 10:**
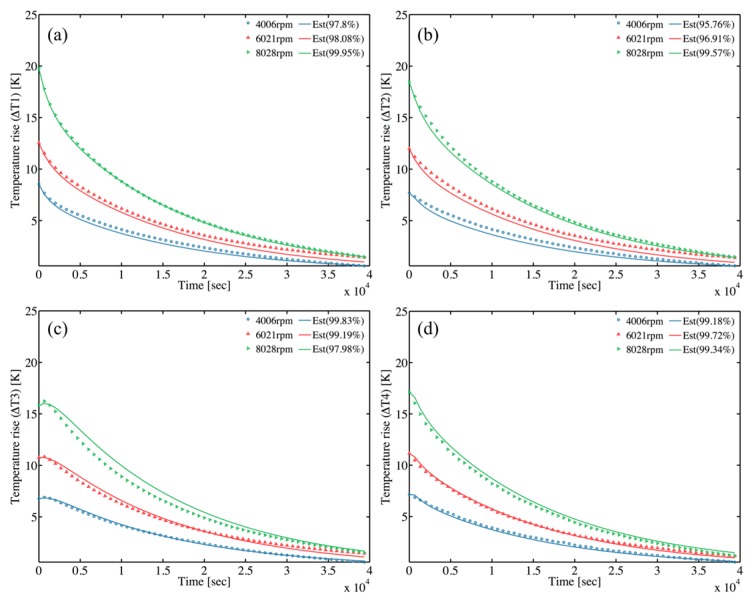
Model self-validation at natural cooling mode with various rotation speeds (**a**) Rear bearing (*T*_1_) temperature validation (**b**) Midpoint of housing (*T*_2_) temperature validation (**c**) Front bearing (*T*_3_) temperature validation (**d**) Midpoint of shaft (*T*_4_) temperature validation.

The grey-box model identification technique is implemented to estimate the remaining parameters of the TNM at both operating and natural cooling modes. The transient self-validations are obtained by comparing the transient temperatures rise estimated by TNM with the measured ones. Five transient states are validated; namely, the spindle runs from stationary to five different steady speeds, say 4006, 5013, 6021, 7023, and 8028 rpm. Figure 9 and Figure 10 show promising validation with the minimum normalized mean square errors (NMSE) of 94.2% at operating mode and 95.8% at natural cooling mode. When the spindle operates at operating mode, the spindle shaft rotates at a constant speed; hence, the temperature probe is not able to contact the shaft. For this reason, the experimental temperature of spindle shaft (*T*_4_) cannot be obtained, and the *T*_4_*-est* represents the estimated shaft temperature, as shown in Figure 9d.

### 5.3. External Validation

**Figure 11:**
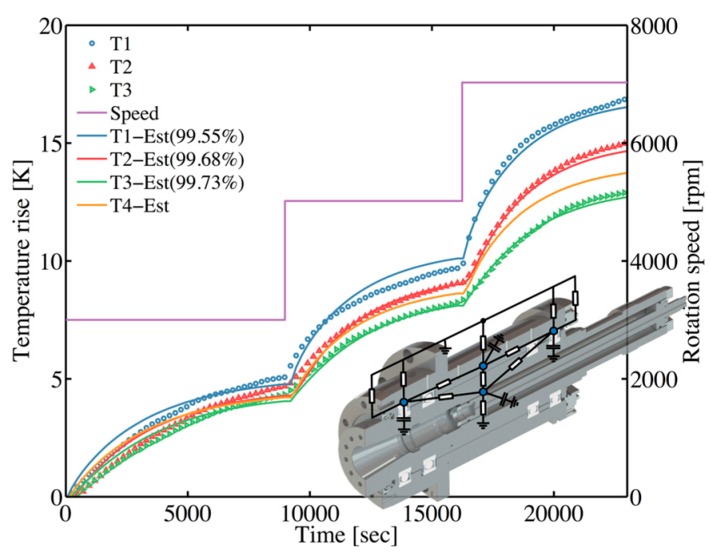
External validation between measured temperature and predicted temperature of estimated TNM under stepwise rotational speed (3001, 5018, 7028 rpm).

To verify the performance and robustness of the TNM, the external validation is necessary. The experiment under stepwise rotational speed (3001, 5018, 7028 rpm) is utilized especially. The results (Figure 11) reveal that the predicted temperature is in satisfactory agreement with the experimental results. As a result, this TNM methodology is appropriate to implement to the machine tool spindle as a simplified thermal model.

### 5.4. Model Order Reduction

**Figure 12:**
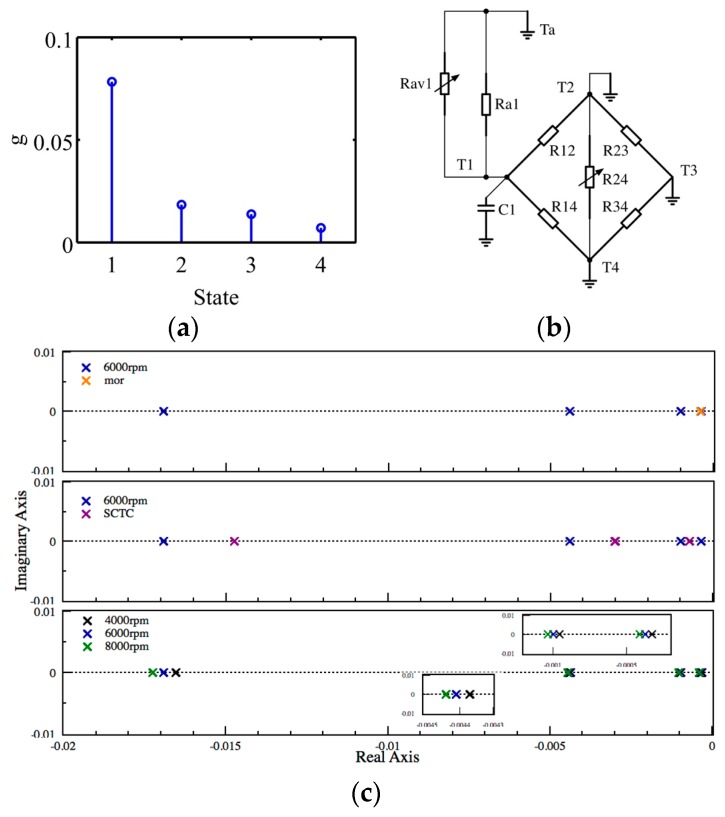
(**a**) Hankel singular values; (**b**) Short circuit time constant method with C2–C4 are short circuited; (**c**) Pole location of estimated TNM when comparing with the Model Order Reduction (MOR) model, SCTC model and varying with rotational speed.

**Figure 13:**
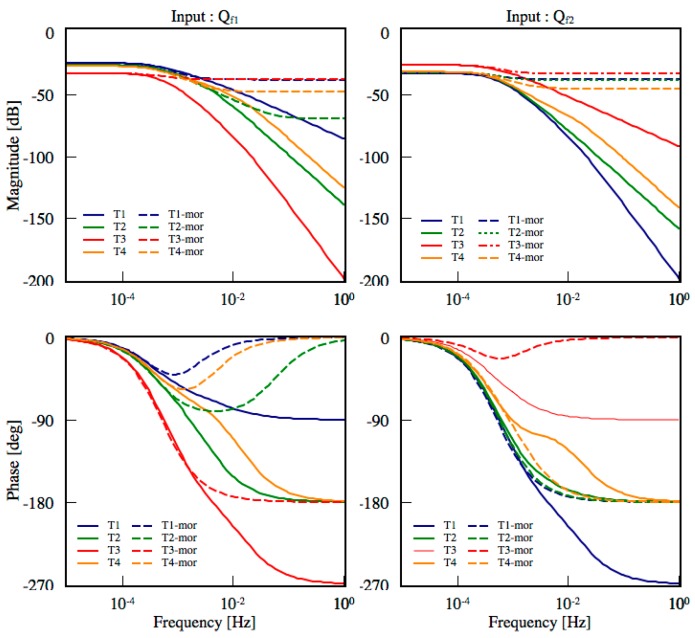
Bode diagram of estimated TNM and 1st-order truncated model based on MOR.

To implement the system parameter estimation into a micro control unit for real-time onboard application on edge computing, the order of TNM model should be further simplified to reduce the load of computation and data storage. MOR technique is an excellent approach for reducing the complexity of the estimated TNM. Balanced realization method is proposed to reduce the 4th-order TNM into 1st-order truncated model, which is a simplified realization with equal controllability and observability. Figure 12a shows the Hankel singular values of the estimated TNM; it points out that there exists a leading state with the larger value than other states. For this reason, we remove the less three states (2, 3, 4) and apply the first dominant state to reconstruct the 1st-order truncated TNM. The pole location of the estimated TNM and truncated model are shown in Figure 12c. In addition, the Bode diagram illustrates the relationship between the input heat generation (*Q_f_*_1_, *Q_f_*_2_) and the output temperature *T*_1_ to *T*_4_ at rotational speed of 6000 rpm. It indicates that the truncated model has equivalent performance to the original estimated TNM under low frequency (<10^−3^) condition, as shown in Figure 13.

### 5.5. Short Circuit Time Constant

The Short Circuit Time Constant (SCTC) method provides an approximation of the poles of the estimated TNM. Each pole can be calculated according to each thermal capacitance multiplied by the nearby equivalent parallel thermal resistance while the other capacitances are short circuited, refer to Figure 12b. As the aspect of system degradation, we can simply recognize the time-varying trend by examining the variation of the time constant instantly by applying SCTC method.

## 6. Conclusions

**Figure 14:**
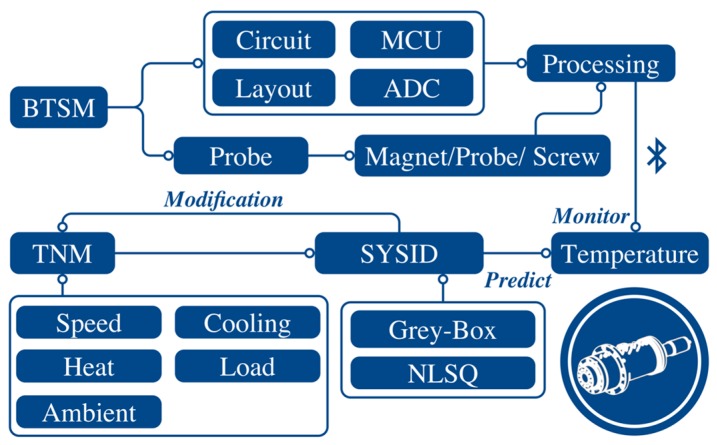
Schematic summary.

**Figure 15:**
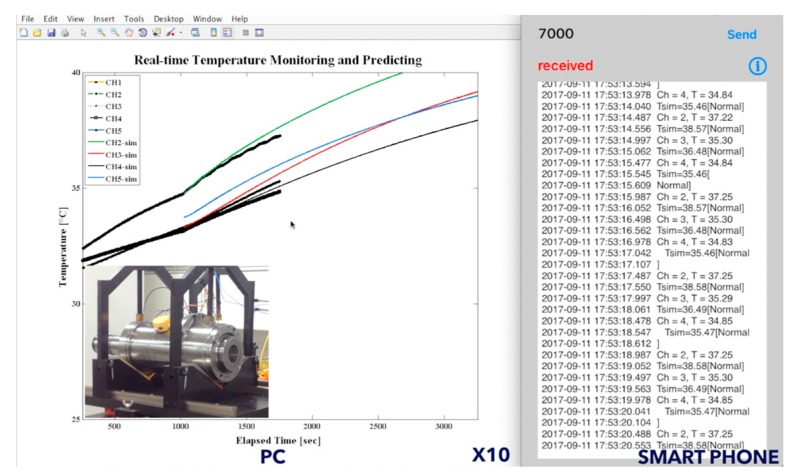
Demonstration of the thermo-feature identification system.

In this article, we investigate the possibility of simplifying the spindle thermal behaviors by estimating the lumped-parameter TNM with system identification technique. We classify the spindle heat transfer condition into two modes, the operating mode and the natural cooling mode. The predicted thermal response of TNM agrees well with the experimental temperature results, verified by self-validation and external validation. In the aspect of the real-time onboard application, the reduced-order model by balanced realization has successfully lowered the computational complexity. In practice, the spindle warm-up procedure is essential to all of the machine tool spindles, especially for the new spindle. By operating at step-wise speeds, the lubricant within the spindle bearings can be evenly spread. The modeling methodology in this research can be appropriately integrated with the warm-up procedure and establish the estimated TNM for further operation. Figure 14 summarizes that the TNM methodology has the ability to predict the spindle thermal behavior; and the BTSM provides real-time monitoring the spindle temperature distribution. In that case, the vision of edge computing within a minimize temperature sensor module can be realized by integrated with the estimated TNM performed as the thermo-feature identification system (TID-sys), which can be customized for each machine tool spindle. Figure 15 demonstrates the real-time user interface of TID-sys on smartphone and PC. On the right-hand side, the TID-sys diagnosis the state (normal or alarm) of the machine tool spindle displayed through the terminal App [32] on smartphone. On the left-hand side, the measured temperature (black dot) of rear bearing (*T*_1_), midpoint of housing (*T*_2_), and front bearing (*T*_3_) attempt to grow along with the curve predicted by the estimated TNM. The TID-sys ensure that the machine tool spindle runs under normal condition. Once the significant temperature variation or abnormal temperature rise is detected, the operator can slow down the spindle or even stop for early protection. The full demonstration video is shown in supplementary materials Video S1.

## Figures and Tables

**Table 1:** BTSM Specification.

Sensor Element	Accuracy [°C]	Resolution [°C]	Measurement Range [°C]	Power [mW]	Module Size [mm^3^]
RTD	±(0.1 + 0.0029|*ϑ*|)	0.00489	−40~150	7	Ø40×

**Table 2:** Estimated thermal parameters of TNM on operating mode.

Parameter	Value	Parameter	Value	Parameter	Value
*R*_12_ [KW^−1^]	0.1263	*R_a_*_1_ [KW^−1^]	0.884	*R_av_*_3_ [KW^−1^]	1.0032
*R*_14_ [KW^−1^]	0.1537	*R_a_*_2_ [KW^−1^]	1.393	*q_f_*_1_ [W]	37.162
*R*_23_ [KW^−1^]	0.5954	*R_a_*_3_ [KW^−1^]	3.071	*q_f_*_2_ [W]	28.406
*R*_24_ [KW^−1^]	3.609	*R_av_*_1_ [KW^−1^]	1.498	*q_f_*_3_ [W]	0.0043
*R*_34_ [KW^−1^]	0.48	*R_av_*_2_ [KW^−1^]	0.303	*q_f_*_4_ [W]	0.0073

**Table 3:** Estimated thermal parameters of TNM on natural cooling mode.

Parameter	Value	Parameter	Value	Parameter	Value
*R*_12_ [KW^−1^]	0.1263	*R*′*_a_*_1_ [KW^−1^]	18.765	*C*_1_ [JK^−1^]	5375.8
*R*_14_ [KW^−1^]	0.1537	*R_a_*_2_ [KW^−1^]	1.393	*C*_2_ [JK^−1^]	3545.8
*R*_23_ [KW^−1^]	0.5954	*R*′*_a_*_3_ [KW^−1^]	6.496	*C*_3_ [JK^−1^]	10,931.7
*R*′_24_ [KW^−1^]	6.737	*R*′*_a_*_4_ [KW^−1^]	2.497	*C*_4_ [JK^−1^]	625.4
*R*_34_ [KW^−1^]	0.48				

## Supplementary Materials

The following are available online at http://www.mdpi.com/1424-8220/18/2/656/s1, Figure S1: Fabrication process of the temperature probe, Figure S2: Experimental setup; and Video S1: Demonstration of the TID-sys.

## Appendix A. System Parameter Matrices

The input, output and state vector of TNM and linear in state space representation:

(A1) $$\begin{array}{l} x={\left[\begin{array}{cccc} \theta_{1} & \theta_{2} & \theta_{3} & \theta_{4} \end{array}\right]}^{T}\\ u={\left[\begin{array}{cccc} Q_{f1} & 0 & Q_{f2} & 0 \end{array}\right]}^{T}\\ y={\left[\begin{array}{cccc} \theta_{1} & \theta_{2} & \theta_{3} & \theta_{4} \end{array}\right]}^{T} \end{array}$$

The heat balance equations of TNM:

(A2) $$\{\begin{array}{c} \frac{-\theta_{1}}{R_{a1}}+\frac{-\theta_{1}}{R_{av1}}+\frac{\theta_{2}-\theta_{1}}{R_{12}}+\frac{\theta_{4}-\theta_{1}}{R_{14}}+Q_{f1}=C_{1}{\dot{\theta }}_{1}\\ \frac{-\theta_{2}}{R_{a2}}+\frac{\theta_{1}-\theta_{2}}{R_{12}}+\frac{\theta_{3}-\theta_{2}}{R_{23}}+\frac{\theta_{4}-\theta_{2}}{R_{24}}=C_{2}{\dot{\theta }}_{2}\\ \frac{-\theta_{3}}{R_{a3}}+\frac{-\theta_{3}}{R_{av2}}+\frac{\theta_{2}-\theta_{3}}{R_{23}}+\frac{\theta_{4}-\theta_{3}}{R_{34}}+Q_{f2}=C_{3}{\dot{\theta }}_{3}\\ \frac{-\theta_{4}}{R_{av3}}+\frac{\theta_{1}-\theta_{4}}{R_{14}}+\frac{\theta_{2}-\theta_{4}}{R_{24}}+\frac{\theta_{3}-\theta_{4}}{R_{34}}=C_{4}{\dot{\theta }}_{4} \end{array}$$

The TNM system matrices of state-space representation at operating mode:

(A3) $$\begin{array}{l} A_{op}=A_{op\_cond}+A_{op\_conv}\\ A_{op\_cond}=\left[\begin{array}{cccc} (-R_{12}^{-1}-R_{14}^{-1})C_{1}^{-1} & R_{12}^{-1}C_{1}^{-1} & 0 & R_{14}^{-1}C_{1}^{-1}\\ R_{12}^{-1}C_{2}^{-1} & (-R_{12}^{-1}-R_{23}^{-1}-R_{24}^{-1})C_{2}^{-1} & R_{23}^{-1}C_{2}^{-1} & R_{24}^{-1}C_{2}^{-1}\\ 0 & R_{23}^{-1}C_{3}^{-1} & (-R_{23}^{-1}-R_{34}^{-1})C_{3}^{-1} & R_{34}^{-1}C_{3}^{-1}\\ R_{14}^{-1}C_{4}^{-1} & R_{24}^{-1}C_{4}^{-1} & R_{34}^{-1}C_{4}^{-1} & (-R_{14}^{-1}-R_{24}^{-1}-R_{34}^{-1})C_{4}^{-1} \end{array}\right]\\ A_{op\_conv}=\left[\begin{array}{cccc} (-R_{a1}^{-1}-R_{av1}^{-1})C_{1}^{-1} & 0 & 0 & 0\\ 0 & -R_{a2}^{-1}C_{2}^{-1} & 0 & 0\\ 0 & 0 & (-R_{a3}^{-1}-R_{av2}^{-1})C_{3}^{-1} & 0\\ 0 & 0 & 0 & -R_{av3}^{-1}C_{4}^{-1} \end{array}\right]\\ B_{op}=diag(C_{1}^{-1},C_{2}^{-1},C_{3}^{-1},C_{4}^{-1});\\ C_{op}=I_{4\times 4};\;D_{op}=O_{4\times 4}; \end{array}$$

The TNM system matrices of state-space representation at steady state:

(A4) $$\begin{array}{l} A_{std}=\left[\begin{array}{cccc} -R_{a1}^{-1}-R_{12}^{-1}-R_{14}^{-1} & R_{12}^{-1} & 0 & R_{14}^{-1}\\ R_{12}^{-1} & -R_{a2}^{-1}-R_{12}^{-1}-R_{23}^{-1} & R_{23}^{-1} & 0\\ 0 & R_{23}^{-1} & -R_{a3}^{-1}-R_{23}^{-1}-R_{34}^{-1} & R_{34}^{-1}\\ R_{14}^{-1} & 0 & R_{34}^{-1} & -R_{14}^{-1}-R_{34}^{-1} \end{array}\right]\\ B_{std}=\left[\begin{array}{cccc} 0 & 0 & 0 & 0\\ 0 & -r_{24}^{-1} & 0 & r_{24}^{-1}\\ 0 & 0 & 0 & 0\\ 0 & r_{24}^{-1} & 0 & -r_{24}^{-1} \end{array}\right];C_{std}=\left[\begin{array}{cccc} -r_{av1}^{-1} & 0 & 0 & 0\\ 0 & 0 & 0 & 0\\ 0 & 0 & -r_{av2}^{-1} & 0\\ 0 & 0 & 0 & -r_{av3}^{-1} \end{array}\right];\\ D_{std}=\left[\begin{array}{ccc} q_{f1} & \cdots & q_{f1}\\ 0 & \cdots & 0\\ q_{f2} & \cdots & q_{f2}\\ 0 & \cdots & 0 \end{array}\right];E_{std}=\left[\begin{array}{ccc} q_{f3} & \cdots & q_{f3}\\ 0 & \cdots & 0\\ q_{f4} & \cdots & q_{f4}\\ 0 & \cdots & 0 \end{array}\right]; \end{array}$$

The TNM system matrices of state-space representation at natural cooling mode:

(A5) $$\begin{array}{l} A_{nc}=A_{nc\_cond}+A_{nc\_conv}\\ A_{nc\_cond}=\left[\begin{array}{cccc} (-R_{12}^{-1}-R_{14}^{-1})C_{1}^{-1} & R_{12}^{-1}C_{1}^{-1} & 0 & R_{14}^{-1}C_{1}^{-1}\\ R_{12}^{-1}C_{2}^{-1} & (-R_{12}^{-1}-R_{23}^{-1}-R_{24}^{\prime -1})C_{2}^{-1} & R_{23}^{-1}C_{2}^{-1} & R_{24}^{\prime -1}C_{2}^{-1}\\ 0 & R_{23}^{-1}C_{3}^{-1} & (-R_{23}^{-1}-R_{34}^{-1})C_{3}^{-1} & R_{34}^{-1}C_{3}^{-1}\\ R_{14}^{-1}C_{4}^{-1} & R_{24}^{\prime -1}C_{4}^{-1} & R_{34}^{-1}C_{4}^{-1} & (-R_{14}^{-1}-R_{24}^{\prime -1}-R_{34}^{-1})C_{4}^{-1} \end{array}\right]\\ A_{nc\_conv}=\left[\begin{array}{cccc} R_{a1}^{\prime -1}C_{1}^{-1} & 0 & 0 & 0\\ 0 & -R_{a2}^{-1}C_{2}^{-1} & 0 & 0\\ 0 & 0 & -R_{a3}^{\prime -1}C_{3}^{-1} & 0\\ 0 & 0 & 0 & -R_{a4}^{\prime -1}C_{4}^{-1} \end{array}\right]\\ B_{nc}=O_{4\times 4};\;C_{nc}=I_{4\times 4};\;D_{nc}=O_{4\times 4}; \end{array}$$

Mean average percentage error:

(A6) $$\mathrm{MAPE}=\frac{1}{n}\sum_{i=1}^{n}\left|\frac{x_{forecast,i}-x_{actual,i}}{x_{actual,i}}\right|\times 100\%$$

Normalized mean square error:

(A7) $$\mathrm{NMSE}=1-\frac{{\left\|x_{ref}-x\right\|}^{2}}{{\left\|x_{ref}-{\bar{x}}_{ref}\right\|}^{2}}$$
